# Point of Care Ultrasound Diagnosis of Pseudoaneurysm of an Upper Extremity Arteriovenous Dialysis Graft 

**DOI:** 10.24908/pocus.v7iKidney.15021

**Published:** 2022-02-01

**Authors:** Forrest Lindsay-Mcginn, Nathaniel C Reisinger

**Affiliations:** 1 Emergency Department, Hospital of the University of Pennsylvania Philadelphia, PA USA; 2 Department of Nephrology, Hospital, University of Pennsylvania Philadelphia, PA USA

**Keywords:** Point-of-Care Ultrasound (POCUS), arteriovenous graft, pseudoaneurysm, dialysis graft

## Abstract

We describe the rapid diagnosis with point of care ultrasound (POCUS) of two acute pseudoaneurysms of a bovine arteriovenous dialysis graft with superimposed cellulitis in a 44-year old male patient who presented with pain over his upper arm graft site. POCUS evaluation decreased the time to diagnosis and vascular surgery consultation.

Point of care ultrasound (POCUS) using a linear transducer revealed two pseudoaneurysms (Figure 1 and 4, online Video S1 and S2) of the graft that were not initially identified on physical exam due to severe tenderness on exam and tissue edema. After informing the patient of this finding, he revealed that he had in fact noticed two painful bumps develop immediately after his last dialyses session. The graft was otherwise patent without thrombus in the lumen. Each pseudoaneurysm appeared as a 4mm and 5mm defect within the graft wall, which is referred to as the neck of the pseudoaneurysm. The neck led to a sac where blood could be seen swirling within the two similarly sized pseudoaneurysms on B-mode. Each measured approximately 1.5 x 3 x 3 cm. Color Doppler showed a pulsating jet entering the pseudoaneurysm from the neck (Figure 1). Additionally, cobblestoning of the subcutaneous tissue was observed directly adjacent to the graft at the proximal aspect, supporting the suspected underlying diagnosis of cellulitis (Figure 2). The patient was treated with vancomycin and cefazolin. POCUS prompted consultation of vascular surgery who removed the graft on day four of admission with a post-operative diagnosis of graft infection. Angiography with computed tomography was performed for preoperative planning (Figure 3). Surgical pathology showed myxoid degeneration of part of the graft wall. Tissue cultures from the graft site taken on the fourth day of antibiotic therapy were negative, including acid-fast bacilli, anerobic bacteria, and fungus. Gram stain was negative. Blood cultures drawn prior to antibiotic administration were negative. Transthoracic echocardiogram showed no valvular vegetations. The patient was discharged after eight days of hospital admission on cefazolin with a tunneled dialysis catheter. 

**Figure 1:**
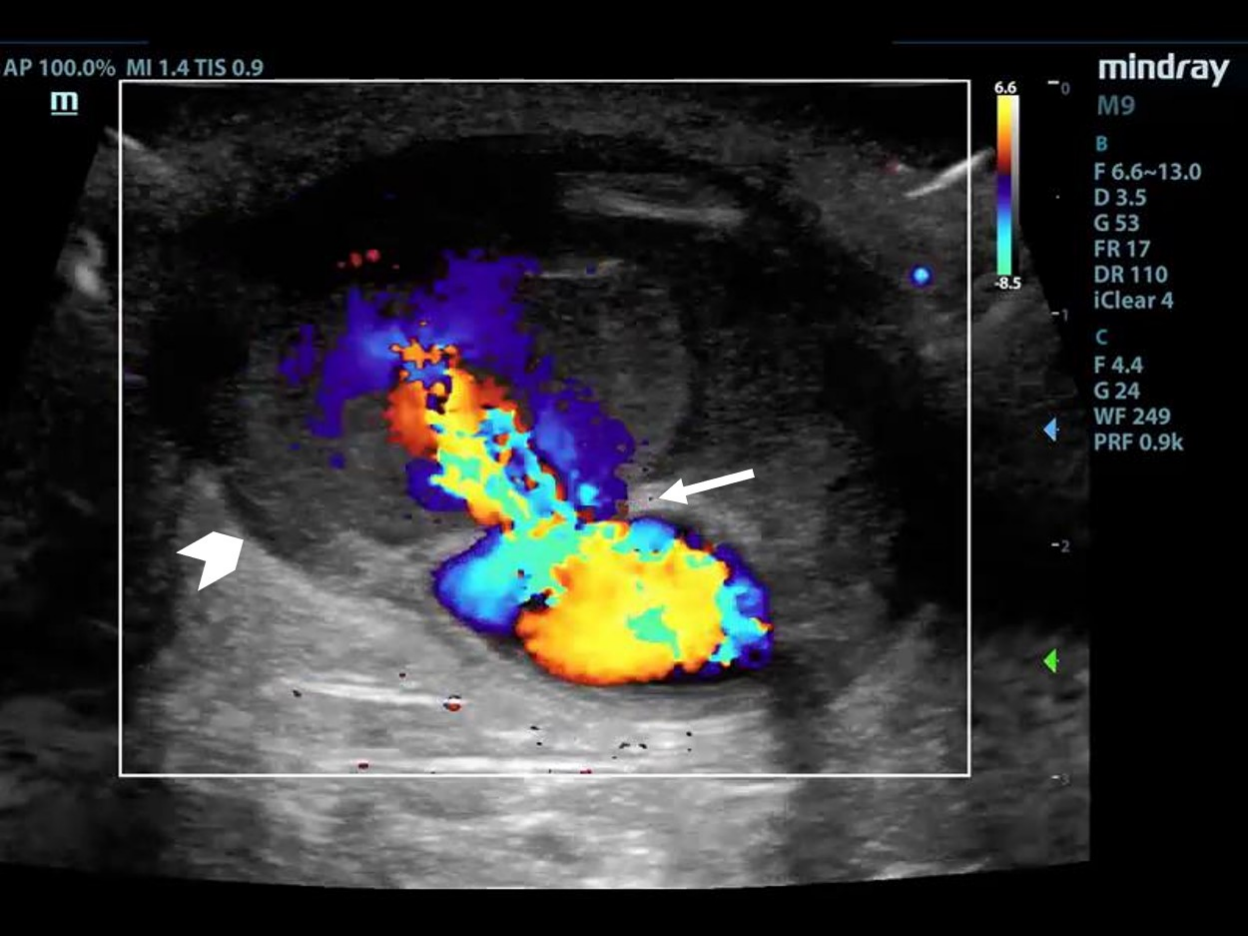
Short axis of AV graft can be seen with pseudoaneurysm neck (arrow) and sac (arrowhead). Color Doppler shows flow in graft and jet into pseudoaneurysm sac. Notice wall of graft is not continuous with wall of sac signifying pseudoaneurysm.

**Figure 2:**
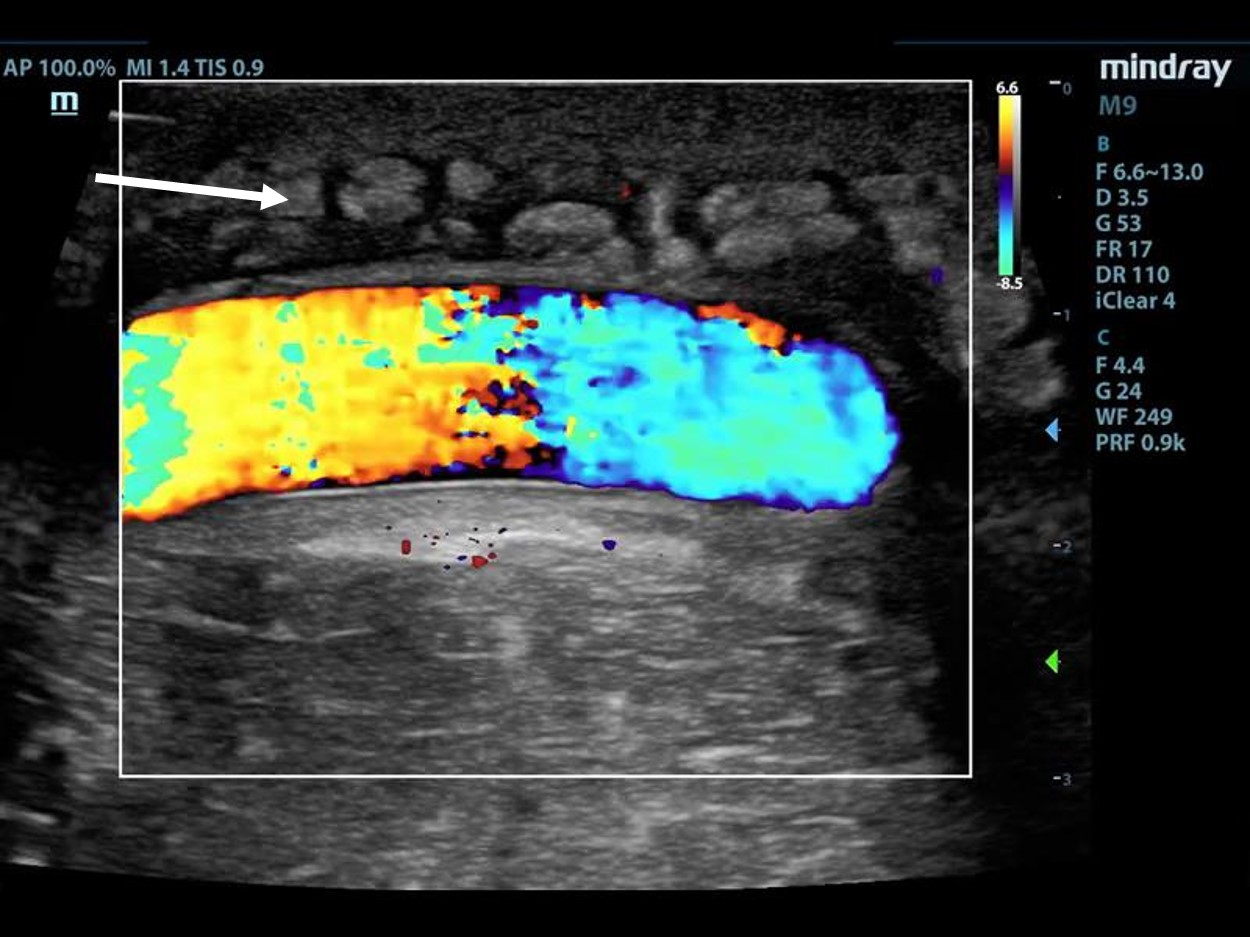
Patent segment of AV graft withcobblestoning (arrow) in the subcutaneous tissue suggestive of cellulitis.

**Figure 3:**
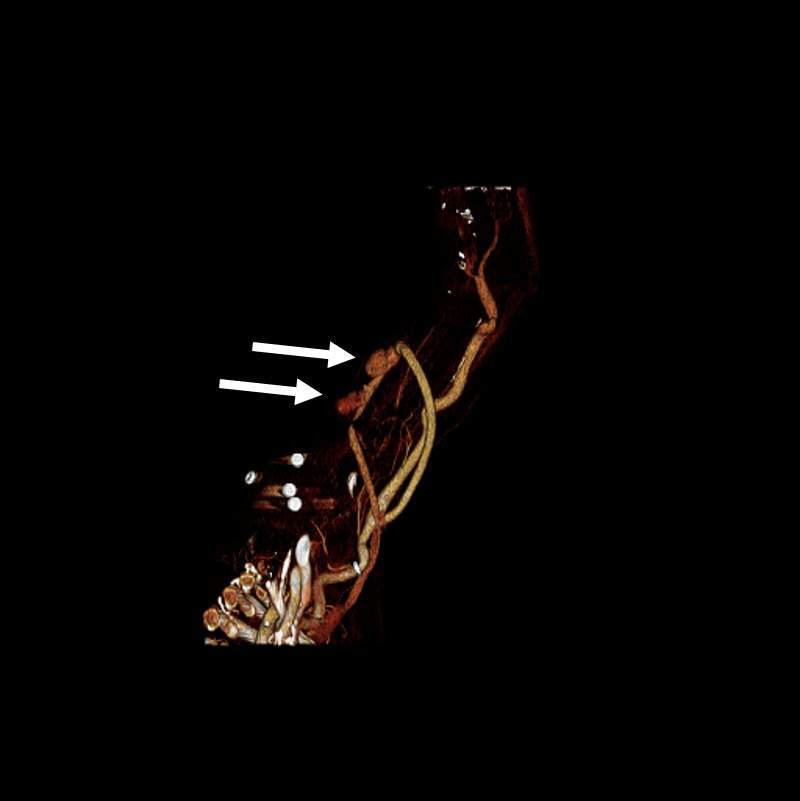
Reconstruction of computed tomography angiography of the left upper extremity with both pseudoaneurysm sacs visible adjacent to AV graft (arrows).

**Figure 4:**
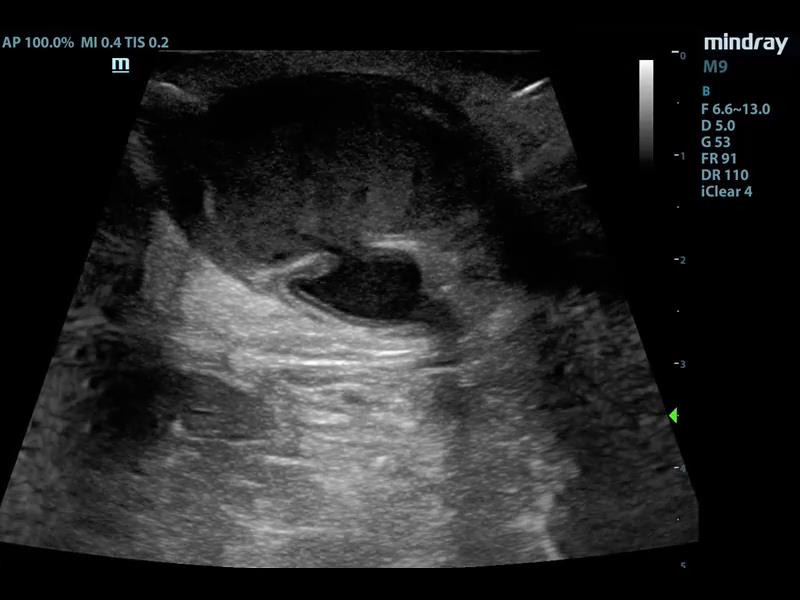
Short axis of AV graft can be seen with pseudoaneurysm neck and sac.

A pseudoaneurysm is sometimes called a false aneurysm because it lacks all three normal elements of the arterial wall. The intima and media are disrupted and the adventitia acts as the outer layer of the pseudoaneurysm, which is why it is sometimes referred to as a contained rupture. The predominate risk is complete rupture, leading to life threatening bleeding or secondary infection. They are often caused by needle puncture and frequently associated with infection 1. Pseudoaneurysm with pending rupture of AV grafts are relatively uncommon, accounting for an estimated 3% of AV graft failure, compared to 13% of AV fistula failure. The most common cause of AV graft failure is thrombosis and infection which account for 80% and 14% respectively 2. Management requires vascular surgery consult for consideration of stent-graft placement or surgical repair with a bypass interposition graft around the site of the pseudoaneurysm. In cases of graft breakdown and infection, ligation or removal may be required. Angiographic examination or ultrasound can be used to assist in surgical planning 3. 

## Disclosures

The patient consented for use of ultrasound images and clinical information for academic purposes. The authors have no conflicts of interest to declare.

## Supplementary Material

 Video S1 Short axis of AV graft can be seen with pseudoaneurysm neck and sac. Color Doppler shows flow in graft and pulsatile flow into sac.

Video S2 Short axis of AV graft can be seen with pseudoaneurysm neck and sac.

## References

[R157094126306630] Jones D W, Farber A, Creager M A, Beckman J A, Loscalzo J (2020).

[R157094126306631] Harms J, Rangarajan S, Young C (2016). Outcomes of arteriovenous fistulas and grafts with or without intervention before successful use. J of Vasc Surg.

[R157094126306632] Patel D V, Vachharajani T J (2018). Principles of treating enlarging pseudoaneurysm in dialysis arteriovenous graft. Hemodialysis Int.

